# Incident Coronary Heart Disease After Preeclampsia: Role of Reduced Fetal Growth, Preterm Delivery, and Parity

**DOI:** 10.1161/JAHA.116.004158

**Published:** 2017-03-06

**Authors:** Hilde Kristin Refvik Riise, Gerhard Sulo, Grethe S. Tell, Jannicke Igland, Ottar Nygård, Stein Emil Vollset, Ann‐Charlotte Iversen, Rigmor Austgulen, Anne Kjersti Daltveit

**Affiliations:** ^1^ KG Jebsen Center for Diabetes Research Department of Clinical Science University of Bergen Norway; ^2^ Department of Global Public Health and Primary Care University of Bergen Norway; ^3^ Department of Health Registries Norwegian Institute of Public Health Bergen Norway; ^4^ Department of Heart Disease Haukeland University Hospital Bergen Norway; ^5^ Centre for Disease Burden Norwegian Institute of Public Health Oslo/Bergen Bergen Norway; ^6^ Department of Cancer Research and Molecular Medicine Centre of Molecular Inflammation Research Norwegian University of Science and Technology (NTNU) Trondheim Norway

**Keywords:** cardiovascular disease, fetal growth restriction, major coronary events, preeclampsia, preterm delivery, Cardiovascular Disease, Epidemiology, Pregnancy, Risk Factors, Women

## Abstract

**Background:**

Preeclampsia is a severe pregnancy disorder often complicated by reduced fetal growth or preterm delivery and is associated with long‐term maternal morbidity and mortality. We aimed to assess the association between preeclampsia phenotypes and risk of subsequent coronary heart disease and maternal cardiovascular mortality.

**Methods and Results:**

Women aged 16 to 49 years who gave birth during 1980–2002 and registered in the Medical Birth Registry of Norway were followed prospectively (1–29 years) for an incident major coronary event and mortality through linkage with the Cardiovascular Disease in Norway 1994–2009 (CVDNOR) project and the Norwegian Cause of Death Registry. Preeclampsia was subdivided based on the presence of a child born small for gestational age or preterm delivery. Among 506 350 women with 1 to 5 singleton births, there were 1275 (0.3%) occurrences of major coronary event, 468 (0.1%) cardiovascular deaths, and 5411 (1.1%) deaths overall. Compared with women without preeclampsia, the hazard ratio (95% CI) for major coronary event was 2.1 (1.73–2.65) after preeclampsia alone, 3.3 (2.37–4.57) after preeclampsia in combination with small for gestational age, and 5.4 (3.74–7.74) after preeclampsia in combination with preterm delivery. Analyses distinguishing women with 1 (n=61 352) or >1 (n=281 069) lifetime pregnancy and analyses with cardiovascular mortality as outcome followed the same pattern.

**Conclusions:**

The occurrence of major coronary events was increased among women with preeclampsia and highest for preeclampsia combined with a child born small for gestational age and/or preterm delivery.

## Introduction

Preeclampsia is a hypertensive pregnancy disorder that is associated with increased morbidity and mortality for both mother and child.^1^ Preeclampsia manifests as hypertension and proteinuria in the latter half of 2% to 8% of all pregnancies, and is more prevalent in the first pregnancy.^2^ No optimal predictive test or efficient treatment for preeclampsia exists. Development of preeclampsia often starts in early pregnancy with reduced remodeling of the uterine spiral arteries and formation of atherosclerosis‐like lesions, and the resulting disturbed blood flow leads to formation of a damaged placenta.^3^ The dysfunctional placenta becomes a stressed organ characterized by oxidative stress and inflammation as the fetus grows and demands a fully functional placenta.^4^ Eventually, the release of antiangiogenic and inflammatory factors to the maternal circulation becomes too high a burden and the mother develops systemic inflammation with endothelial dysfunction, thrombophilia, and impaired hemodynamics.^5^, ^6^ Similar processes also characterize development of coronary heart disease.^7^ A close connection between development of preeclampsia and later cardiovascular disease (CVD) has become apparent in recent years,^8^ but how these vascular diseases are connected is still not clear.

In addition to being a heterogeneous disorder with clinical signs ranging from mild to life‐threatening, preeclampsia is often interrelated with adverse pregnancy outcomes such as reduced fetal growth and preterm delivery, with partly shared underlying pathogenic mechanisms and risk factors.^9^, ^10^ These complications are all independent risk factors for subsequent maternal CVD.^11^, ^12^, ^13^ For improved insight into the relationship between preeclampsia and CVD, a broader look at related pregnancy complications and CVD should be undertaken. We have identified 3 studies in which the relationship between preeclampsia, reduced fetal growth, preterm delivery, and subsequent cardiovascular morbidity risk has been investigated.^14^, ^15^, ^16^ Preeclampsia and gestational hypertension were shown to be associated with subsequent development of ischemic heart disease, and the risk was highest for preeclampsia combined with reduced fetal growth and/or preterm delivery. However, these studies: (1) did not focus on acute coronary events but did include stable angina pectoris; (2) covered a limited time span following pregnancy; (3) did not fully assess parity; and (4) examined smaller cohorts (2 studies^15^, ^16^).

By linking data from the Medical Birth Registry of Norway (MBRN) to the nationwide Cardiovascular Disease in Norway 1994–2009 (CVDNOR) project (www.cvdnor.no) containing data on all CVD hospitalizations and deaths, we aimed to examine whether preeclampsia alone or combined with reduced fetal growth and/or preterm delivery increases the risk of subsequent major coronary events (MACEs) and cardiovascular mortality. In addition, we wanted to examine whether the association varies according to number of lifetime pregnancies.

## Material and Methods

### Data Sources

Information on 708 614 women aged 16 to 49 years at childbirth with a first birth registered in the MBRN during 1980–2009 was obtained. MBRN is a nationwide mandatory registry containing information on all pregnancies above 16 weeks' gestation in Norway since 1967. Data from the antenatal forms are transferred to the MBRN after delivery. The MBRN provides data for epidemiological surveillance of maternal health, pregnancy complications, birth defects, and other perinatal outcomes, and for research on their causes and consequences.^17^

To follow women prospectively for MACE, we linked the MBRN to the CVDNOR project, the Norwegian Cause of Death Registry, Statistics Norway, and the National Population Registry, from January 1980 through December 2009. The CVDNOR project contains information on all hospitalizations from all somatic hospitals in Norway with a CVD or diabetes‐related discharge diagnosis from 1994 to 2009.^18^ Underlying cause and date of death were obtained from the Norwegian Cause of Death Registry. From Statistics Norway and the National Population Registry, information on sociodemographic status, date of death, and date of emigration were extracted.

### Exclusions

**Figure 1:**
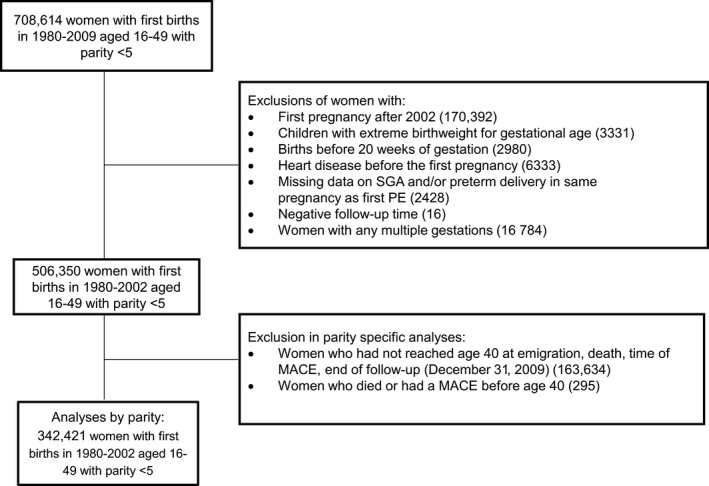
Flow diagram of number of study patients. Data on 708 614 women with a first birth registered in the Medical Birth Registry of Norway during 1980–2009 were available. MACE indicates major coronary event; PE, preeclampsia; preterm delivery, <37 weeks of gestation; SGA, small for gestational age (<10th percentile).

In order to have at least 7 years of observation time after a woman's first childbirth, to look for subsequent deliveries, women with their first birth after 2002 were excluded (170 392), leaving 538 222 women. Women with any births before gestational week 20 (n=2980) were excluded, since preeclampsia is diagnosed after 20 weeks of gestation. Based on diagnosis in the MBRN or the CVDNOR project, 6333 women with existing heart disease at first delivery were excluded (*International Classification of Diseases, Tenth Revision* [*ICD‐10*]: I01, I03–09, I11, I13, I20–25, I30–52 [and corresponding for *ICD‐8* and *ICD‐9*]). Women with babies with a *z* score outside (−4 +4) of birthweight by gestational week (n=3331) or missing data on small for gestational age (SGA) and/or preterm delivery in the same pregnancy as the first incidence of preeclampsia (n=2428) were excluded. Women with any multiple gestation were excluded from the analyses (n=16 784). Sixteen women with negative follow‐up time probably due to an erroneous date of death were also excluded. This left us with 506 350 women for the overall analyses (where parity was not considered) (Figure 1). During the study period, 23 472 (4.6%) women emigrated. We conducted additional analyses where we excluded women with a diagnosis of diabetes (defined as type 1, type 2, or unspecified diabetes) before her first pregnancy (n=1727), since diabetes is strongly associated with preeclampsia, preterm birth,^19^ and coronary heart disease.^20^

In parity‐specific analyses, we classified women as having 1 or >1 lifetime pregnancy by the age of 40 years and analyzed the association between preeclampsia and MACE separately. This age cutoff was chosen based on the observation that the majority of women had completed their reproductive career by that age, thus avoiding classifying a woman's number of lifetime pregnancies before she has completed her reproductive career, ie, avoiding immortal time bias. Women younger than 40 years at emigration, death, time of MACE, or end of follow‐up (December 31, 2009) were excluded from analyses (n=163 929). This left us with 342 421 women, of whom 61 352 had 1 birth and 281 069 had >1 lifetime birth. Because preeclampsia is related to the pregnancy, we also use the terms “one lifetime pregnancy” and “more than one lifetime pregnancy,” although the woman may have experienced pregnancies ending in an abortion and hence was not reported in the birth registry.

### Study Exposure and End Points

The MBRN defines preeclampsia as maternal blood pressure of at least 140 mm Hg systolic or 90 mm Hg diastolic, or an increase of >15 mm Hg in blood pressure measured before gestational week 20, in combination with proteinuria (protein excretion >0.3 g per 24 hours or >+1 on dipstick). These criteria are in accordance with the recommendations of the American College of Obstetricians and Gynecologists.^21^ The diagnostic validity of preeclampsia in the MBRN is high.^22^ A broad phenotype was investigated with preeclampsia in the presence or absence of a child born SGA and delivery before or after 36 completed weeks of gestation (<37 or ≥37 weeks). SGA was defined as fetal growth <10th percentile of Norwegian birth weight curves.^23^ A composite exposure variable was generated on the presence of preeclampsia alone or in combination with a child born SGA and/or preterm delivery. Mothers without preeclampsia in any pregnancy served as the reference group. We conducted additional analyses applying more restrictive cutoffs for preterm delivery (<34 weeks) and SGA (<7.5th percentile of Norwegian birth weight curves).

The MACE (main end point) was defined as nonfatal acute myocardial infarction (*ICD‐9*: 410 or *ICD‐10*: I21, I22) or coronary death (*ICD‐9*: 410–414 or *ICD‐10*: I20–I25). Secondary end points included CVD (*ICD‐9*: 390–9; *ICD‐10*: I00–I99) and all‐cause mortality.

In the analyses stratified by parity, we assessed whether exposure‐outcome associations differed according to a woman's lifetime number of births. Women with >1 lifetime birth were stratified into: (1) no preeclampsia at all; (2) preeclampsia in first pregnancy only; (3) preeclampsia in first and second pregnancy; and (4) preeclampsia in second or later but not first pregnancy.

Informed consent was waived because data were collected from national registries. The study was approved by the Regional Committee for Medical and Health Research Ethics (2014/1047).

### Statistical Methods

Data were analyzed by Cox proportional hazard regression on two sets of analyses: (1) all pregnancies starting follow‐up at first birth; and (2) parity‐specific analyses starting follow‐up at age 40. The level of significance was defined as <0.05 in all analyses.

In the overall analysis (n=506 350), associations between exposure categories and all 3 end points were examined using maternal age as the time scale in both Cox regression and in Kaplan–Meier curves. Follow‐up time was calculated as the difference between age at the date of hospital admission, death, emigration or December 31, 2009 (whichever occurred first), and age at first birth. In these analyses, we compared the risk of MACE among women with and without preeclampsia at the same age. Because preeclampsia could occur in any pregnancy, preeclampsia was introduced in the model as a time‐dependent covariate. This was done to avoid classifying the mother as exposed while still unexposed, ie, avoiding immortal time bias.^24^ By using age as a time‐scale and preeclampsia as a time‐dependent covariate we account for the age at which the woman has her first occurrence of preeclampsia, and the age at which the woman has her first MACE.

In the parity‐specific analyses (n=342 421) in‐depth analyses with age as the time scale were done with MACE as the end point and age 40 as the start of follow‐up. Follow‐up time was calculated as the difference between age at the date of hospital admission, death, emigration or December 31, 2009 (whichever occurred first), and age 40. Because 40 years of age was used as the start of follow‐up and preeclampsia exposure was defined retrospectively, women with no preeclampsia before age 40 were treated as unexposed in the Cox regression analyses even if they developed the syndrome after age 40 (n=405). The results of additional analyses reclassifying these women as “exposed” at the time of preeclampsia yielded similar results to those reported here.

For all analyses, potential confounders were evaluated and chosen based on their association with the exposure and end points. Education was classified into 3 categories: basic education (compulsory education), upper secondary education (high school or vocational school), and tertiary education (college or university). Birth year of the first child independent of significance level was included in the model to account for potential cohort effects. Exclusion of women with chronic renal disease before pregnancy (n=8905 [1.8%] in the overall analyses) did not alter the hazard ratios (HRs), and women with chronic renal disease were thus included in all presented analyses. Two‐way interaction terms for all combinations of preeclampsia, education, marital status, and birth year of first child were added in the multiple Cox regression analyses. No significant interactions were found and results are based on models without interaction terms.

## Results

### Characteristics of the Study Population

The characteristics of the 506 350 women in the total study population stratified by parity and preeclampsia diagnosis are shown in Table 1. Information on maternal age and year of birth was complete. Information on maternal education was missing in 1.0% of the women. Gestational week was missing in 1 or more pregnancies in 6.5% of the women. Less than 0.2% of the women had missing values on the baby's birth weight in any pregnancy. Women with missing data were excluded from all analyses.

**Table 1:** Characteristics of 506 350 Norwegian Women With 1 to 5 Singleton Deliveries and a First Delivery During 1980–2002, by Parity and Presence of Preeclampsia

Characteristics of the Study Participants	One Lifetime Pregnancy		More than 1 Lifetime Pregnancy	
	Without Preeclampsia (n=86 180)	With Preeclampsia (n=4385)	Without Preeclampsia (n=390 253)	With Preeclampsia^a^ (n=25 532)
Age at first birth, mean (SD), y	27.5 (5.7)	28.1 (5.6)	25.1 (4.2)	25.0 (4.2)
Education level, No. (%)^b^				
Basic education	30 727 (35.7)	1661 (37.9)	113 396 (29.1)	7531 (29.5)
Secondary education	24 162 (28.0)	1329 (30.3)	118 591 (30.4)	7998 (31.3)
Tertiary education	28 747 (33.4)	1333 (30.4)	155 756 (39.9)	9877 (38.7)
Marital status at first birth, No. (%)^c^				
Married/cohabitant	62 303 (72.3)	3419 (78.0)	325 468 (83.4)	64 785 (16.6)
Other	23 877 (27.7)	966 (22.0)	21 525 (84.3)	4007 (15.7)
Diabetes mellitus, No. (%)^d^	401 (0.5)	117 (2.7)	944 (0.2)	265 (1.0)
Preterm delivery, No. (%)^e^	5292 (6.1)	1080 (24.6)	35 714 (9.2)	6485 (25.4)
Small for gestational age, No. (%)^f^	11 487 (13.3)	1221 (27.8)	65 691 (16.8)	7631 (29.9)
Study end points, No. (%)				
Major coronary event	352 (0.4)	55 (1.3)	743 (0.2)	125 (0.5)
Cardiovascular deaths	171 (0.2)	21 (0.5)	256 (0.1)	20 (0.1)
All deaths	2034 (2.4)	109 (2.5)	3065 (0.8)	203 (0.6)
Follow‐up time until major coronary event, median (IQR), y	17.2 (11.3)	15.9 (11.7)	18.5 (10.8)	17.8 (11.0)
Follow‐up time until cardiovascular death, median (IQR), y	17.2 (11.3)	15.7 (11.6)	18.5 (10.8)	17.8 (11.0)

IQR indicates interquartile range.

a In at least 1 of the pregnancies.

b Information on education was not available in 5242 (1.04%) of women .

c Information on cohabitant only available from 1982.

d Diabetes mellitus diagnosed before the first pregnancy.

e Less than 37 weeks of gestation.

f Lower than the 10th percentile.

The majority of the mothers had >1 lifetime birth (82.1%). Women with only 1 lifetime birth were less likely to be married/cohabitants, were older at the time of giving birth, and had lower education and a higher prevalence of prepregnancy diabetes.

Among the 29 917 (5.9%) women who had preeclampsia in at least 1 pregnancy, 21 635 (72.3%) had a preeclamptic first pregnancy. A preeclamptic first pregnancy occurred among 4385 (4.8%) women with only 1 lifetime birth and among 17 250 (4.1%) women with >1 lifetime birth. Preeclamptic women were twice as likely to have a child born SGA (13.3% versus 27.8% for women with 1 lifetime pregnancy and 16.8% versus 29.9% for women with >1 lifetime pregnancy) and 3 times more likely to deliver preterm (6.1% versus 24.6% for women with one lifetime pregnancy and 9.2% versus 25.4% for women with >1 lifetime pregnancy) compared with women without preeclampsia (Table 1).

During follow‐up, 1275 (0.3%) women experienced MACEs and 468 (0.1%) mothers died due to CVD and 5411 (1.1%) due to any cause.

### Preeclampsia and Risk of Subsequent MACE

**Figure 2:**
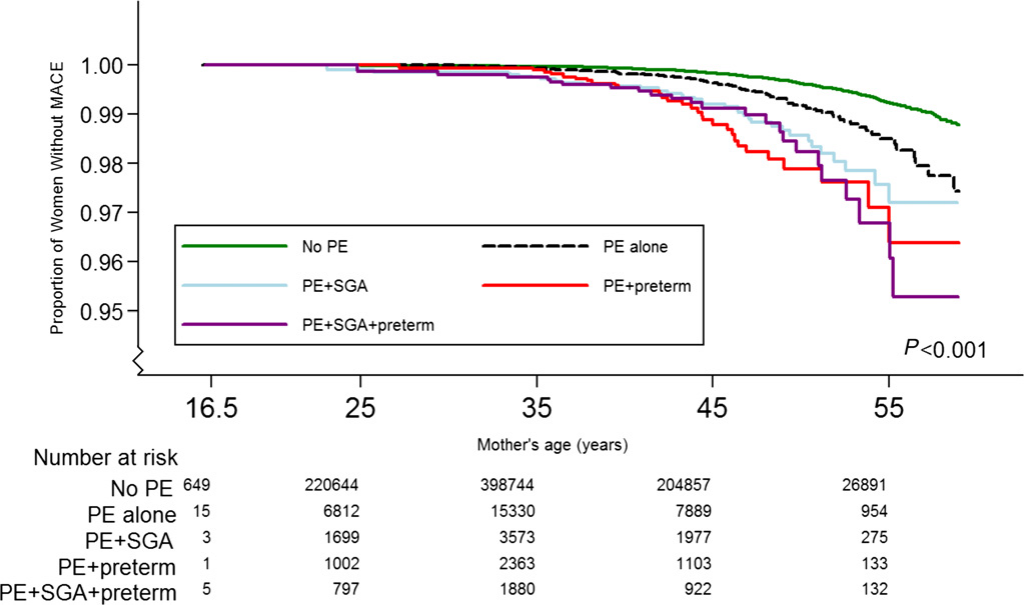
Kaplan–Meier curves of subsequent risk of major coronary events (MACEs) according to preeclampsia (PE) status (*P*<0.001). A total of 506 350 women aged 16 to 49 years were included and 1275 MACEs (0.3%) were registered. Preterm delivery indicates <37 weeks of gestation; SGA, small for gestational age (<10th percentile).

The Kaplan–Meier curve shows the difference in survival between women without preeclampsia compared with those with preeclampsia only, preeclampsia combined with SGA, preterm delivery, or both (*P*<0.001) (Figure 2).

The associations between preeclampsia and subsequent MACEs and cardiovascular mortality are shown in Table 2. Women who experienced preeclampsia alone showed a doubled risk for subsequent MACEs compared with women without preeclampsia, and this increased risk remained statistically significant after adjusting for potential confounders (HR, 2.1; 95% CI, 1.73–2.65). Women with preeclampsia combined with SGA and/or preterm delivery showed a further doubled risk of MACEs (HR, 4.3; 95% CI, 2.81–6.43) compared with women with preeclampsia only. The risk for MACE was highest for women with preeclampsia in combination with preterm delivery (HR, 5.38; 95% CI, 3.74–7.74) (Table 2).

**Table 2:** Preeclampsia and Subsequent Risk of Major Coronary Events and Cardiovascular Mortality Among 506 350 Norwegian Women With 1 to 5 Singleton Deliveries and a First Delivery During 1980–2002

Study End Points	No./Events	Unadjusted		Adjusted^a^	
		HR (95% CI)	*P* Value	HR (95% CI)	*P* Value
Major coronary event					
No preeclampsia	476 506/1095	1	···	1	···
Preeclampsia only	19 617/90	2.17 (1.75–2.69)	<0.001	2.14 (1.73–2.65)	<0.001
Preeclampsia+SGA and/or preterm delivery^b^	10 227/90	4.18 (3.37–5.18)	<0.001	4.05 (3.27–5.02)	<0.001
Preeclampsia+SGA	4495/37	3.49 (2.51–4.84)	<0.001	3.30 (2.37–4.57)	<0.001
Preeclampsia+preterm delivery	3266/30	5.25 (3.65–7.55)	<0.001	5.38 (3.74–7.74)	<0.001
Preeclampsia+SGA+preterm delivery	2466/23	4.42 (2.93–6.68)	<0.001	4.25 (2.81–6.43)	<0.001
Cardiovascular mortality					
No preeclampsia	476 506/427	1	···	1	···
Preeclampsia only	19 617/26	1.62 (1.09–2.41)	0.017	1.60 (1.08–2.38)	0.020
Preeclampsia+SGA and/or preterm delivery	10 227/26	3.13 (1.09–2.41)	<0.001	3.06 (2.06–4.54)	<0.001
Preeclampsia+SGA	4495/16	3.97 (2.41–6.54)	<0.001	3.72 (2.26–6.13)	<0.001
Preeclampsia+preterm delivery	3266/6	2.64 (1.18–5.91)	0.018	2.81 (1.25–6.29)	0.012
Preeclampsia+SGA+preterm delivery	2466/4	2.00 (0.75–5.35)	0.168	1.93 (0.72–5.16)	0.191

Cox regression analysis with preeclampsia as a time‐dependent covariate. HR indicates hazard ratio; SGA, small for gestational age (<10th percentile).

a Analyses adjusted for education, marital status, and birth year of first child.

b <37 weeks of gestation.

Results from analyses stratified by parity are summarized in Table 3. Among women with only 1 lifetime birth, having preeclampsia only or preeclampsia with SGA and/or preterm delivery increased the risk of subsequent MACEs 2 or 3 times, respectively, compared with women without preeclampsia.

**Table 3:** Preeclampsia and Risk of Subsequent Major Coronary Events Among 342 421 Norwegian Women With 1 to 5 Singleton Deliveries and a First Delivery During 1980–2002, Followed From the Age of 40

	No./Events	Unadjusted		Adjusted^a^	
		HR (95% CI)	*P* Value	HR (95% CI)	*P* Value
All women (N=342 421)					
No preeclampsia	323 323/857	1	···	1	···
Preeclampsia only	12 592/64	2.05 (1.59–2.65)	<0.001	2.02 (1.56–2.60)	<0.001
Preeclampsia+SGA and/or preterm delivery^b^	6506/59	3.76 (2.88–4.89)	<0.001	3.63 (2.79–4.72)	<0.001
One lifetime pregnancy (n=61 352)					
No preeclampsia	58 520/292	1	···	1	···
Preeclampsia only	1663/19	2.36 (1.47–3.76)	<0.001	2.26 (1.42–3.60)	0.001
Preeclampsia+SGA and/or preterm delivery	1169/19	3.56 (2.24–5.66)	<0.001	3.32 (2.09–5.29)	<0.001
More than 1 lifetime pregnancy (n=281 069)					
No preeclampsia	264 803/565	1	···	1	···
Preeclampsia complicating any pregnancy^c^					
Preeclampsia only	10 929/45	2.02 (1.49–2.73)	<0.001	1.97 (1.45–2.67)	<0.001
Preeclampsia+SGA and/or preterm delivery	5337/40	3.85 (2.79–5.31)	<0.001	3.75 (2.72–5.17)	<0.001
Preeclampsia complicating first pregnancy only					
Preeclampsia only	6318/25	1.98 (1.33–2.96)	0.001	1.95 (1.31–2.91)	0.001
Preeclampsia+SGA and/or preterm delivery	2881/16	2.94 (1.79–4.83)	<0.001	2.81 (1.70–4.61)	<0.001
Preeclampsia complicating first and second pregnancies					
Preeclampsia only	937/5	2.31 (0.96–5.58)	0.062	2.20 (0.91–5.32)	0.079
Preeclampsia+SGA and/or preterm delivery	826/8	4.43 (2.21–8.91)	<0.001	4.66 (2.31–9.37)	<0.001
Preeclampsia complicating second or later pregnancies					
Preeclampsia only	3674/15	1.98 (1.19–3.31)	0.009	1.93 (1.16–3.23)	0.012
Preeclampsia+SGA and/or preterm delivery	1630/16	5.11 (3.11–8.39)	<0.001	4.92 (2.99–8.10)	<0.001

Cox regression analyses stratified by parity. HR indicates hazard ratio; SGA, small for gestational age (<10th percentile).

a Adjusted for education, marital status and birth year of first child.

b Preterm delivery (<37 weeks of gestation), SGA, or both.

c The first time preeclampsia occurred, independent of which pregnancy.

Among women with >1 lifetime birth, the risk of MACEs after preeclampsia in the first pregnancy only, was 2.0‐fold (95% CI, 1.31–2.91) for preeclampsia alone and 2.8‐fold (95% CI, 1.70–4.61) for preeclampsia with SGA and/or preterm delivery (Table 3). For women with recurrent preeclampsia the presence of preeclampsia alone doubled the risk of MACE (HR, 2.2; 95% CI, 0.91–5.32) and when combined with SGA and/or preterm delivery the risk was markedly higher at 4.7‐fold (95% CI, 2.31–9.37) compared with women without preeclampsia (Table 3). We tested for interaction between preeclampsia and mode of delivery by doing a likelihood ratio test comparing models with and without an interaction term and found no significant interaction; the association between preeclampsia and MACEs does not vary by mode of delivery (*P*=0.127).

Among women who did not experience preeclampsia, women with 1 lifetime pregnancy had a higher risk of MACEs compared with women with >1 lifetime pregnancy (HR, 1.3; 95% CI, 1.11–1.51).

The difference between analysis with and without diabetes was most pronounced for MACEs after preeclampsia in combination with preterm delivery, where the number of MACEs declined from 30 to 20 and HR declined from 5.4 (95% CI, 3.74–7.74) to 3.9 (95% CI, 2.51–6.09) (Table S1).

We repeated the analyses, applying a cutoff of 34 weeks for preterm delivery and <7.5th percentile for SGA offspring and this suggested stronger associations between MACEs and preeclampsia in combination with SGA and/or preterm delivery. For preeclampsia+preterm delivery, HR increased from 5.4 (95% CI, 3.74–7.74) to 7.0 (95% CI, 4.18–11.5) (Table S2).

### Preeclampsia and Risk of Cardiovascular and All‐Cause Mortality

Women with only preeclampsia showed an increased risk of CVD mortality (HR, 1.6; 95% CI, 1.08–2.38) compared with women without preeclampsia (Table 2). Mortality further increased in women with preeclampsia combined with a child born SGA (HR, 3.7; 95% CI, 2.26–6.13) or preterm delivery (HR, 2.8; 95% CI, 1.25–6.29).

Women with only preeclampsia did not show increased all‐cause mortality, but for preeclamptic women with a SGA offspring and preterm delivery, the risk of dying was 21% to 23% higher than that in women without preeclampsia; however, these results were statistically nonsignificant.

## Discussion

In this large register‐based study, an association was found between preeclampsia and subsequent risk of MACE and cardiovascular mortality. The risk of MACE and cardiovascular mortality was further markedly increased when preeclampsia was combined with a child born SGA and/or preterm delivery, and the highest risk was found for preeclamptic women with preterm delivery. In parity analyses, the highest risk of MACE was found for women with recurrent preeclampsia combined with an SGA offspring and/or preterm delivery.

### Preeclampsia and Subsequent Risk of MACE

Our findings are in line with previous studies linking preeclampsia with subsequent ischemic heart disease.^14^, ^15^, ^16^ It is unclear what leads to this increased risk of MACEs, and several hypotheses have been suggested.^1^, ^8^ Women may have a shared unfavorable cardiovascular and preeclampsia risk profile before pregnancy involving risk factors such as obesity, hypertension, high blood lipids, diabetes, and physical inactivity.^2^ Women may have pleiotropic genetic risk factors that predispose them to develop preeclampsia in pregnancy and CVD later in life.^3^, ^25^ The relationship may at least partly be due to conditions in pregnancy since vascular load of pregnancy complications, such as preeclampsia, may create a maternal permanent metabolic or vascular imbalance ultimately resulting in cardiovascular complications. Women undergo major changes in cardiovascular function during pregnancy.^26^ CVD is a result of a complex interplay of multiple factors that span the life course,^27^ and pathways leading to preeclampsia may share common physiologic processes with the development of CVD and MACEs, such as dyslipidemia.

### Preeclampsia in Combination With a Child Born SGA and/or Preterm Delivery

Pregnancy with an SGA offspring and/or preterm delivery further increased the risk of MACEs in women with preeclampsia. Severity of preeclampsia has been shown to be associated with an increased risk of coronary heart disease, although with lower magnitude than for preeclampsia combined with an SGA offspring and/or preterm delivery.^14^ Early development of preeclampsia may lead to preterm delivery, and this form of preeclampsia may have different causes, severity, and outcome compared with preeclampsia manifesting at gestation. The heterogeneous nature of preeclampsia includes individual components of the initial placental disease and of later maternal response to insufficient placentation or even to pregnancy itself, and presence of an SGA offspring may reflect a more severe placental disease of the syndrome.^28^ Such differences between preeclampsia phenotypes were also apparent from the findings that preeclampsia with a child born SGA was more strongly associated with cardiovascular mortality, while preeclampsia with preterm delivery was more strongly associated with MACEs in our study.

### Parity

The relationship between parity and risk of coronary heart disease in women has been assessed in previous studies.^29^ A study from Norway focused on preeclampsia and the risk of cardiovascular mortality comparing women with 1 lifetime pregnancy and women with >1 lifetime pregnancy.^30^ The authors of this study reported the highest cardiovascular mortality risk after preeclampsia in women with only 1 lifetime pregnancy. They also reported a strong effect on cardiovascular mortality of having only 1 child among women without preeclampsia (HR, 2.0). While we found the same HR for mortality (2.1), the HR for MACE was only 1.3. Furthermore, the increase in risk after preeclampsia was similar for women with 1 lifetime pregnancy and women with >1 lifetime pregnancy. In our study, the highest risk for MACEs was found for women with recurrent preeclampsia complicating the first 2 pregnancies. This is in accordance with the risk for ischemic heart disease after recurrent preeclampsia in a Danish^14^ and a Swedish study.^16^ Several preeclamptic pregnancies might increase the burden on the cardiovascular system and add to a woman's risk of later CVD.^7^, ^31^, ^32^

### Strengths and Limitations

The strengths of this study include the large nationwide cohort and historical observation of incident cardiovascular events, both fatal and nonfatal. Prevalence of preeclampsia in a woman's first pregnancy has previously been reported to be around 3.6% in Norway,^33^ compared with 4.3 in our study. Reproductive history and information on both parity and gestational age were available and loss to follow‐up was minimal.

Before 1999, only information on pregnancies lasting ≥16 weeks was included in the MBRN. Hence, we cannot account for potential effects of early abortions. Early medical interruptions of normal pregnancies may reduce the risk of preeclampsia in a subsequent pregnancy due to immunological changes, while spontaneous abortions can, to a larger extent, be associated with other factors, such as infertility, that may increase the risk of preeclampsia.^34^ Data on subclassification of preeclampsia (mild, severe, unspecified) were only available after 1999 in the MBRN, and instead of subclassifying preeclampsia, a broader approach of addressing the related disorders reduced fetal growth and preterm delivery was chosen to strengthen the study. Inclusion of deliveries from 1980 to 1993 gave up to 14 years without morbidity follow‐up. However, the majority of coronary events occur after age 50 and the median age at first delivery was 25.5 years. For women with first deliveries in 1994, only 4 had incident coronary events the following 10 years and 21 events were registered 10 to 14 years after delivery. Few CVD events are therefore expected before 1994 for women with first deliveries during 1980–1993 and full follow‐up on deaths and serious CVD events were available in this period. Exclusion of women younger than 40 years in the second set of analyses might have introduced selection bias because we restricted the analyses to a population older than 40 years without any MACEs before the age of 40. We calculated that the annual incidence of acute myocardial infarction among women aged 25 to 39 years in Norway between 2001–2009 was low (from 11 to 15 per 100 000 persons per year). Hence, the exclusion of these events was unlikely to substantially influence our findings. Since our study was not designed to analyze time trends in morbidity, we were not able to determine whether time trends in CVD risk in affected women differed from those in unaffected women. The data from our study are from a period where preventive measures based on risk assessment of women with preeclampsia were not initiated in Norway, and few preeclamptic women in our cohort were offered follow‐up to assess CVD risk and reduce morbidity. Information on underlying CVD risk factors such as smoking and body mass index is missing in MBRN before 1998 because of a lack of registered lifestyle factors.^35^, ^36^, ^37^, ^38^

## Conclusions

Our results demonstrate that development of preeclampsia increases the maternal risk of nonfatal or fatal coronary events and CVD mortality later in life. The increased risk associated with preeclampsia was observed both in mothers with only 1 and in those with >1 lifetime pregnancies. In the latter group, the increased risk for future adverse events was not related to timing of preeclampsia in relation to parity (ie, preeclampsia in the first versus preeclampisa in later pregnancies). When associated with preterm delivery and/or SGA babies, the risk of future adverse coronary events associated with preeclampsia was even higher. Our findings provide evidence in favor of monitoring these women who are at increased risk for future coronary events.

## Sources of Funding

First author Hilde Kristin Refvik Riise, MSN, received a scholarship from “Extrastiftelsen, Nasjonalforeningen for folkehelsen,” Oslo, Norway.

## Disclosures

None.

## Supporting information


**Table S1.** Preeclampsia and Subsequent Risk of Major Coronary Events Among 504 623 Women With 1 to 5 Singleton Deliveries and a First Delivery During 1980–2002 After Exclusion of Women With a Diagnosis of Diabetes Mellitus.* Cox regression analysis with preeclampsia as a time‐dependent covariate.
**Table S2.** Preeclampsia and Subsequent Risk of Major Coronary Events Among 506 397 Women With 1 to 5 Singleton Deliveries and a First Delivery During 1980–2002 With a Lower Cutoff Point for Small for Gestational Age and Preterm Delivery. Cox regression analysis with preeclampsia as a time‐dependent covariate.
