# Supplementary material for: Incident Coronary Heart Disease After Preeclampsia: Role of Reduced Fetal Growth, Preterm Delivery, and Parity
Source: J Am Heart Assoc. 2017 Mar 6;6(3):e004158. doi: 10.1161/JAHA.116.004158 (PMC5523993; doi:10.1161/JAHA.116.004158)
Supplement: Supplementary file 1 — Table S1. Preeclampsia and Subsequent Risk of Major Coronary Events Among 504 623 Women With 1 to 5 Singleton Deliveries and a First Delivery During 1980–2002 After Exclusion of Women With a Diagnosis of Diabetes Mellitus.* Cox regression analysis with preeclampsia as a time‐dependent covariate. Table S2. Preeclampsia and Subsequent Risk of Major Coronary Events Among 506 397 Women With 1 to 5 Singleton Deliveries and a First Delivery During 1980–2002 With a Lower Cutoff Point for Small for Gestational Age and Preterm Delivery. Cox regression analysis with preeclampsia as a time‐dependent covariate. [file JAH3-6-e004158-s001.pdf]

SUPPLEMENTAL MATERIAL

Table S1. Preeclampsia and Subsequent Risk of Major Coronary Events Among 504,623 Women With 1 to 5 Singleton Deliveries and a First Delivery During 1980–2002 After Exclusion of Women With a Diagnosis of Diabetes Mellitus*

		Unadjusted		Adjusted†	
	No./Events	HR (95% CI)	P value	HR (95% CI)	P value
**Major coronary event**					
No preeclampsia	475 160/1063	1	…	1	…
Preeclampsia only	19 417/83	2.07 (1.66–2.59)	<0.001	2.05 (1.64–2.56)	<0.001
Preeclampsia + SGA and/or preterm delivery‡	10 046/76	3.68 (2.92–4.65)	<0.001	3.58 (2.84–4.52)	<0.001
Preeclampsia + SGA	4480/35	3.39 (2.42–4.75)	<0.001	3.21 (2.29–4.49)	<0.001
Preeclampsia + preterm delivery	3123/20	3.77 (2.42–5.87)	<0.001	3.91 (2.51–6.09)	<0.001
Preeclampsia + SGA + preterm delivery	2442/21	4.19 (2.72–6.45)	<0.001	4.05 (2.62–6.24)	<0.001

Cox–regression analysis with preeclampsia as a time–dependent covariate. HR indicates hazard ratio; CI, confidence interval; SGA, small for gestational age (<10th percentile).

* Diabetes mellitus occurring prior to first pregnancy.

† Adjusted for education, marital status and birth year of first child.

‡ <37 weeks of gestation.

Table S2. Preeclampsia and Subsequent Risk of Major Coronary Events Among 506,397 Women With 1 to 5 Singleton Deliveries and a First Delivery During 1980–2002 With a Lower Cutoff Point for Small For Gestational Age and Preterm Delivery

		Unadjusted		Adjusted†	
	No./Events	HR (95% CI)	P value	HR (95% CI)	P value
**Major coronary event**					
No preeclampsia	476 506/1095	1	…	1	…
Preeclampsia only	22 815/114	2.39 (1.97-2.90)	<0.001	2.36 (1.94-2.86)	<0.001
Preeclampsia + SGA and/or preterm delivery‡	7056/66	4.26 (3.32-5.46)	<0.001	4.09 (3.19-5.25)	<0.001
Preeclampsia + SGA	4886/41	3.59 (2.62-4.90)	<0.001	3.41 (2.50-4.66)	<0.001
Preeclampsia + preterm delivery	1265/15	6.77 (4.07-11.27)	<0.001	6.96 (4.18-11.59)	<0.001
Preeclampsia + SGA + preterm delivery	925/10	5.40 (2.89-10.07)	<0.001	5.13 (2.75-9.57)	<0.001

Cox–regression analysis with preeclampsia as a time–dependent covariate. HR indicates hazard ratio; CI, confidence interval; SGA, small for gestational age (<7.5th percentile).

† Adjusted for education, marital status and birth year of first child.

‡ <34 weeks of gestation.
